# Restoration of NAD^+^ homeostasis protects C2C12 myoblasts and mouse levator ani muscle from mechanical stress-induced damage

**DOI:** 10.1080/19768354.2022.2106303

**Published:** 2022-08-03

**Authors:** Guotao Huang, Yong He, Li Hong, Min Zhou, Xiaohu Zuo, Zhihan Zhao

**Affiliations:** a Department of Gynecology and Obstetrics, Renmin Hospital of Wuhan University, Wuhan, Hubei Province, People’s Republic of China

**Keywords:** Mechanical stress, levator ani muscle, C2C12 myoblast, Oxidized nicotinamide adenine dinucleotide, mitochondrial dysfunction

## Abstract

Excessive mechanical traction damages the levator ani muscle (LAM), increasing the incidence of pelvic floor dysfunction (PFD). In this study, we explored the effects of oxidized nicotinamide adenine dinucleotide (NAD^+^) on the damage to both muscle cells and LAM tissue induced by mechanical stress (MS) at the cellular and animal levels. The cell damage model was established using a four-point bending system. The LAM damage model was established using vaginal distention and traction. Exogenous addition of PJ34, an inhibitor of poly (ADP-ribose) polymerase-1 (PARP-1), and the nicotinamide mononucleotide (NMN) precursor of NAD^+^ increased NAD^+^ levels. ATP content and mitochondrial membrane potential were measured to assess mitochondrial function. NAD^+^ levels, cell viability, and PARP-1 activity were detected using commercial kits. DNA damage in cells was detected with immunofluorescence staining, and LAM damage was detected with tissue TUNEL staining. PARP-1 activity and DNA damage of LAM were detected by immunohistochemistry. A small amount of DNA damage and PARP-1 activation did not affect NAD^+^ levels, while excessive DNA damage and PARP-1 activation led to an imbalance of NAD^+^ homeostasis. Furthermore, increasing NAD^+^ levels *in vivo* and *in vitro* could rescue mitochondrial dysfunction and damage to both muscle cells and LAM tissue induced by MS. In conclusion, MS can induce damage to both C2C12 cells and LAM tissue. Restoring NAD^+^ homeostasis can rescue this damage by improving mitochondrial function.

## Introduction

Pelvic floor dysfunction (PFD) refers to a variety of disorders related to moderate to severe impairment of the pelvic floor muscles. According to epidemiological investigations, the prevalence of PFD in adult women is 20–40% (Erekson et al. 2015; Islam et al. 2016). Research on the pathogenesis and treatment of PFD has important clinical and social implications. Damage to pelvic floor tissues, such as LAM, can lead to the occurrence of PFD (DeLancey 1997). Acute mechanical damage caused by vaginal delivery and chronic mechanical damage caused by obesity, constipation, and pregnancy are risk factors for PFD (Sangsawang 2014). Excessive mechanical traction caused by pregnancy, childbirth, and elevated abdominal pressure can lead to LAM damage, which increases the incidence of PFD (Kearney et al. 2006; Bozkurt et al. 2014). Therefore, studying the mechanism of LAM injury induced by MS is important to explore the pathogenesis of PFD.

Mitochondria are the energy factories of cells that affect cellular environmental homeostasis in various ways (Wallace 2012). Mitochondrial dysfunction can lead to cell metabolism disorders and even cell death. The dysfunction is closely related to the occurrence of many diseases, including cardiovascular disease, neurodegeneration, and neuromuscular disease (Sorrentino et al. 2018). Similarly, pathological changes occur in the mitochondria in the pelvic floor tissue of PFD patients (Han et al. 2014; Yiou et al. 2009). In addition, our previous study demonstrated that excessive MS induces myoblast mitochondrial damage (Yi et al. 2020). NAD^+^ is a hydrogen/electron transfer molecule involved in the metabolism of fatty acids and glucose. It is essential for the maintenance of mitochondrial function and plays an important role in muscle development, homeostasis, and various diseases (Goody and Henry 2018). NAD^+^ redox imbalance and mitochondrial dysfunction were observed in a pressure overload myocardial model. Exogenous NAD^+^ supplementation through the salvage synthesis pathway can significantly improve mitochondrial and myocardial function (Lee et al. 2016). However, the role of NAD^+^ in MS-induced LAM injury remains unclear.

Our previous study showed that MS could cause an oxidation-antioxidant imbalance in LAM tissues and cause the accumulation of reactive oxygen species (ROS) in cells (Yi et al. 2020). Excessive accumulation of ROS can cause gaps or breaks in the DNA strands (Ishikawa et al. 2008). Poly (ADP-ribose) polymerase1 (PARP-1) catalyzes its own activity by recognizing gaps or breaks in the DNA strands and participates in a variety of biological reactions using NAD^+^ as a substrate. Continuous an extensive DNA damage leads to the overactivation of PARP-1, which reduces or even depletes NAD^+^ (Chambon et al. 1963; Pascal 2018; Virag et al. 1998; Martin-Guerrero et al. 2020). Excessive mechanical stretching of human bronchial epithelial cells can induce oxidative stress and DNA damage, and increase PARP-1 activity (Wang et al. 2014).

We speculated that MS could induce DNA damage, activate PARP-1, and deplete NAD^+^, leading to mitochondrial dysfunction and even cell death. As the samples of human LAM tissue were difficult to obtain, we only studied the effects of NAD^+^ on the damage to muscle cells and LAM tissue induced by MS at the cellular and animal levels.

## Material and Methods

### Cell culture and treatments

C2C12 mouse myoblasts were obtained from Nanjing Kezhen Biotechnology Co. Ltd. (Nanjing, China). The cells were maintained in Dulbecco’s modified Eagle’s medium (Genom, Hangzhou, China) containing 100 μg/mL streptomycin, 100 U/mL penicillin G (Genom, Hangzhou, China), and 10% fetal bovine serum. Cells were placed in an incubator (HEALFOR CE, Hongkong, China) at 37 °C in an atmosphere of 5% CO_2_. To evaluate the effect of NMN supplementation, C2C12 cells were treated with vehicle (PBS) or 500 μM NMN (MACKLIN, Shanghai, China) for 24 h after applying MS. To inhibit PARP-1, C2C12 cells were treated with vehicle (PBS) or 1 μM PJ34 hydrochloride (MedChemExpress, Monmouth Junction, NJ, USA) for 24 h before MS.

### Establishment of cell damage model induced by MS

The model was based on our previous study (Yi et al. 2020). When the cell density reached 70–80%, the cell strain culture plates were transferred to the strain loading dish, which was placed in the control power system for MS. MS experiments were performed using a four-point bending device (Chengdu Power Technology Co., Ltd., Chengdu, China). The loading parameters were set to 2666 μ (the deformation displacement of the culture plate was 2 mm) for 2 h (MS1), 2666 μ for 4 h (MS3), 2666 μ for 8 h (MS5), 5333 μ (the deformation displacement of the culture plate was 4 mm) for 2 h (MS2), 5333 μ for 4 h (MS4), and 5333 μ for 8 h (MS6) at a frequency of 1 Hz.

### DNA damage of C2C12 cells

As DNA damage can induce phosphorylation of H2A histone family member X (H2AX), DNA damage in C2C12 cells was detected by immunofluorescence staining of phospho-H2AX (pH2AX). After washing with PBS, C2C12 cells were fixed with 4% paraformaldehyde for 15 min and permeabilized with 0.25% Triton X-100 in PBS for 15 min. Cells were then blocked with PBS containing 0.01 g/mL bovine serum albumin for 60 min and then incubated with pH2AX antibody solution for 60 min. Finally, cells were incubated with Alexa Fluor 555 goat anti-rabbit IgG and Hoechst33342 for 60 min in the dark. The experiments were conducted at room temperature. Stained cells were imaged using a model CKX31 fluorescence microscope (Olympus Corporation, Tokyo, Japan). The nucleus was fluoresced blue and pH2AX fluoresced red.

### PARP-1 activity of C2C12 cells

PARP-1 activity in C2C12 cells was detected using the PARP-1 Activity Fluorescence Quantitative Detection Kit (Product number: GMS50494.1; GenMed Scientifics Inc., Wilmington, DE, USA) according to the manufacturer’s instructions. After being lysed, the cell samples were sequentially added to buffer (GenMed Scientifics Inc.), reaction solution (GenMed Scientifics Inc.), and substrate solution (GenMed Scientifics Inc.), and incubated in the dark at room temperature for 20 min. Then, alkaline solution (GenMed Scientifics Inc.) and color developing solution (GenMed Scientifics Inc.) were sequentially added prior to incubation in the dark at 4°C for 10 min. Samples then received an acid solution (GenMed Scientifics Inc.) and were incubated in an oven at 110°C for 5 min. After cooling at room temperature for 15 min, the samples were measured with a microplate reader.

### Cell viability

Cell viability was detected using the Cell Counting Kit-8 (CCK8) kit (Product number: C0037; Beyotime Institute of Biotechnology, Shanghai, China) according to the manufacturer’s instructions.

### Animal experimental design and treatments

Wild-type C57BL/6 female mice (9–10 weeks old, 20–26 g, virgin) were obtained from the Animal Experiment Center of Wuhan University (Wuhan, China). This study was approved by the Ethics Committee of the Institutional Animal Care and Use Committee of the Renmin Hospital, Wuhan University (approval number: 20210306). The establishment of the LAM injury model induced by MS was based on our previous study (Yi et al. 2020). Briefly, virgin mice underwent vaginal distention (balloon distention volume of 0.4 mL) and traction (VDT). The mice that had the uninflated balloon alone comprised the sham group and untreated mice comprised the control group. To evaluate the effect of different traction forces and traction time, the traction parameters were set to 20 g for 0.5 h (VDT1), 20 g for 1 h (VDT3), 20 g for 2 h (VDT5), 40 g for 0.5 h (VDT2), 40 g for 1 h (VDT4), and 40 g for 2 h (VDT6). To evaluate the effect of NMN supplementation, the mice received injections (intraperitoneally) of vehicle (PBS) or 500 mg NMN/kg body weight for 7 consecutive days after undergoing VDT. To inhibit PARP-1, the mice were intraperitoneally injected with vehicle (PBS) or 10 mg PJ34/kg body weight for 24 h before undergoing VDT. On day 8 after VDT, the mice were sacrificed, and the LAM was harvested.

### NAD^+^ measurement

NAD^+^ in LAM and C2C12 cells was quantified using an NAD^+^/NADH detection kit (Product number: S0175; Beyotime Institute of Biotechnology) according to the manufacturer’s instructions. Cells and tissues were lysed using a special extraction solution. After centrifugation, the supernatant was used as the sample for testing. The sample was added directly to a 96-well plate for total NAD^+^/NADH detection. After being heated in a 60°C water bath for 30 min to remove NAD^+^, the sample was used to detect NADH content. After being added to the alcohol dehydrogenase solution, the sample was incubated in the dark at 37°C for 10 min, followed by addition of color developing solution and incubation in the dark at 37°C for 30 min. The NADH content was determined using a microplate reader.

### Mitochondrial function

Adenosine 5’-triphosphate (ATP) content in LAM and C2C12 cells was measured using the Enhanced ATP Assay Kit (Beyotime Institute of Biotechnology). (Product number: S0027) according to the manufacturer’s instructions. For ATP detection, the cells and tissues were lysed with lysis buffer. After centrifugation, the supernatant was used as the sample for testing. The samples was added to the test solution and immediately measured using a microplate reader. Mitochondrial membrane potential (ΔΨm) from LAM and C2C12 cells was measured using an assay kit (Product number: C2006; Beyotime Institute of Biotechnology) according to the manufacturer’s instructions. Cell samples were each resuspended in JC-1 staining solution and incubated at 37°C for 20 min. After centrifugation, the cells were washed twice with the staining buffer. Finally, the samples were analyzed using a microplate reader. For tissue samples, mitochondria of LAM tissue were extracted using the Tissue Mitochondria Isolation Kit (Product number: C3606; Beyotime Institute of Biotechnology). The resuspended mitochondria was added to JC-1 staining solution and analyzed using a microplate reader.

### Immunohistochemistry

Immunohistochemistry was performed as previously described (Yi et al. 2020). Poly (ADP-ribosylation) (PAR, Novus Biologicals, US. Product number: NBP2-89039) and gamma H2A.X (p S139) (γH2AX, Servicebio, Wuhan, China, Product number: GB111841) were used as the primary antibody in the experimental protocol. Nuclei of cells were stained blue with hematoxylin. Positive staining of diaminobenzidine (DAB) was brownish yellow.

### Terminal deoxynucleotidyl transferase dUTP nick end labeling (TUNEL) staining of tissue

After deparaffinization, rehydration, and antigen retrieval, tissue samples were incubated with the TUNEL solution for 2 h in the dark. Subsequently, each tissue was washed three times with PBS and incubated with 4′,6-diamidino-2-phenylindole (DAPI) solution for 10 min in the dark. Stained tissues were imaged using fluorescence microscopy. Nuclei stained with DAPI were blue under ultraviolet light excitation, the TUNEL kit was labeled with FastStart Universal SYBR Green Master (Rox), and apoptotic nuclei were green.

### Statistical analyses

Data analyses were performed using SPSS (IBM, Armonk, NY, USA), GraphPad Prism (GraphPad, San Diego, CA, USA), and ImageJ (NIH, Bethesda, MD, USA) software. Statistical data are expressed as the mean ± standard deviation. One-way analysis of variance was used to analyze the differences among the groups. Differences between the two groups were determined using Dunnett’s *t*-test. Tukey’s test was used for multiple comparisons. At least three independent replicate experiments were performed. *P* < 0.05 indicated statistical significance.

## Results

### Mechanical overload causes breakage of DNA strands, increases PARP-1 activity, and reduces levels of NAD^+^ in C2C12 cells

**Figure 1.**
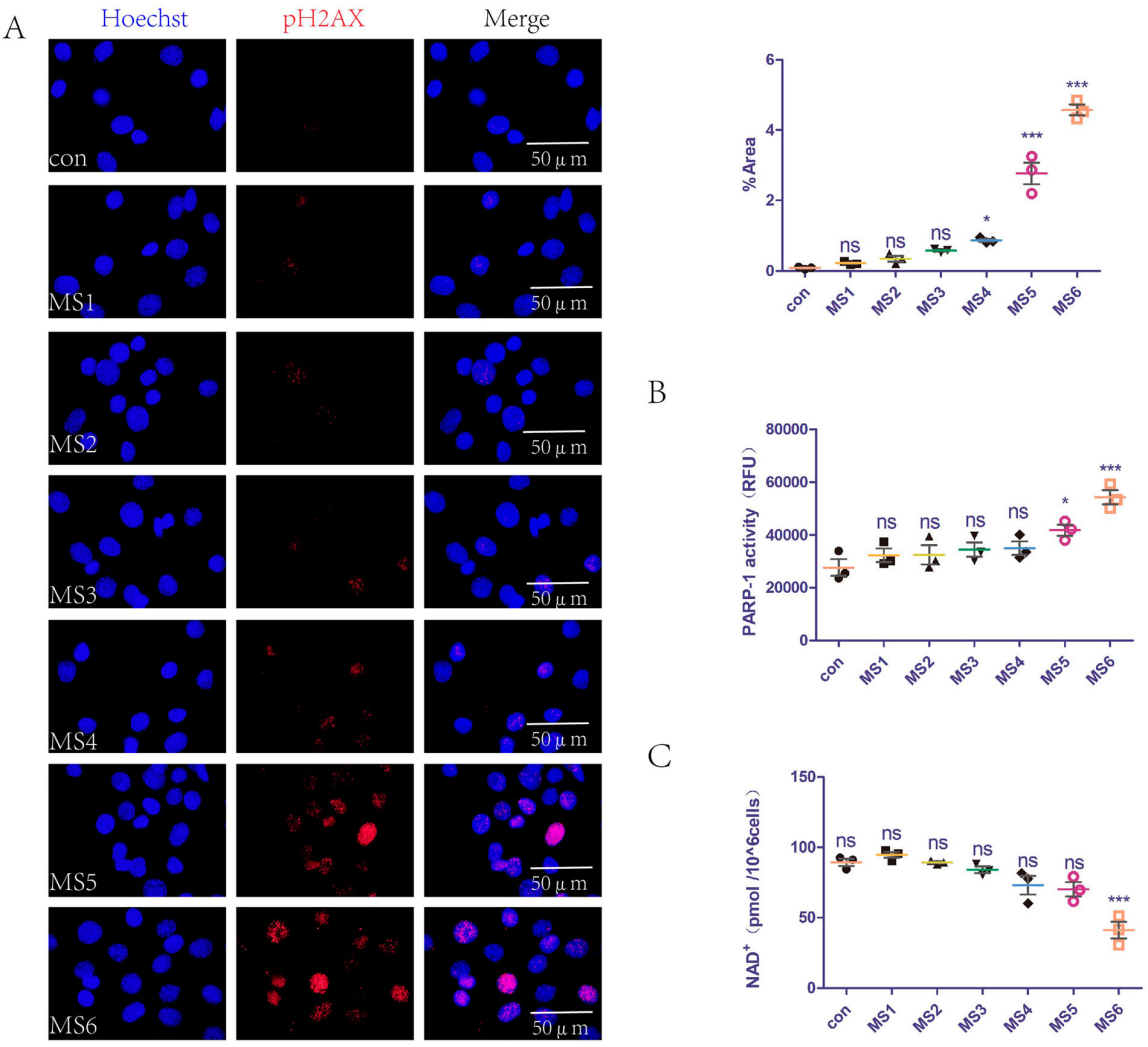
Mechanical overload causes DNA strands break, increases PARP-1 activity, and reduces levels of NAD^+^ in C2C12 cells. (A) DNA damage of C2C12 cells was detected with immunofluorescence staining of pH2AX. Nuclei fluoresced blue and pH2AX fluoresced red. The positive staining area was semi-quantified using Image J software. (B) PARP-1 activity of C2C12 cells detected with a commercial kit. (C) NAD^+^ levels of C2C12 cells detected with a commercial kit. The experimental groups are detailed in the Material and Methods section. All experiments were independently repeated at least three times. The statistical data are expressed as the mean ± standard deviation. Differences between the two groups were determined using Dunnett’s *t*-test. Tukey’s test was performed for multiple comparison (compared with control group: ***p < 0.001, **p < 0.01; *p < 0.05; ns, not significant). RFU denotes relative fluorescence unit.

We previously demonstrated that MS can lead to the accumulation of ROS in cells (Yi et al. 2020), which can cause DNA damage. The present study extended these findings by examining the effect of MS on DNA at different loading parameters. The pH2AX level is a common indicator of DNA damage. The mechanical loading parameters of MS1-3 did not cause DNA damage, while those of MS4-6 did (Figure 1A). In addition, we explored the effects of MS on PARP-1 activity and levels of NAD^+^ in C2C12 cells. The mechanical loading parameters of MS1-4 did not affect PARP-1 activity, while those of MS5-6 caused PARP-1 overactivation (Figure 1B). Furthermore, the MS1-5 mechanical loading parameters did not affect the intracellular NAD^+^ levels, while those of MS6 reduced intracellular NAD^+^ levels (Figure 1C).

### Inhibition of PARP-1 activity rescues cell damage induced by MS by increasing NAD^+^ levels and improving mitochondrial dysfunction

**Figure 2.**
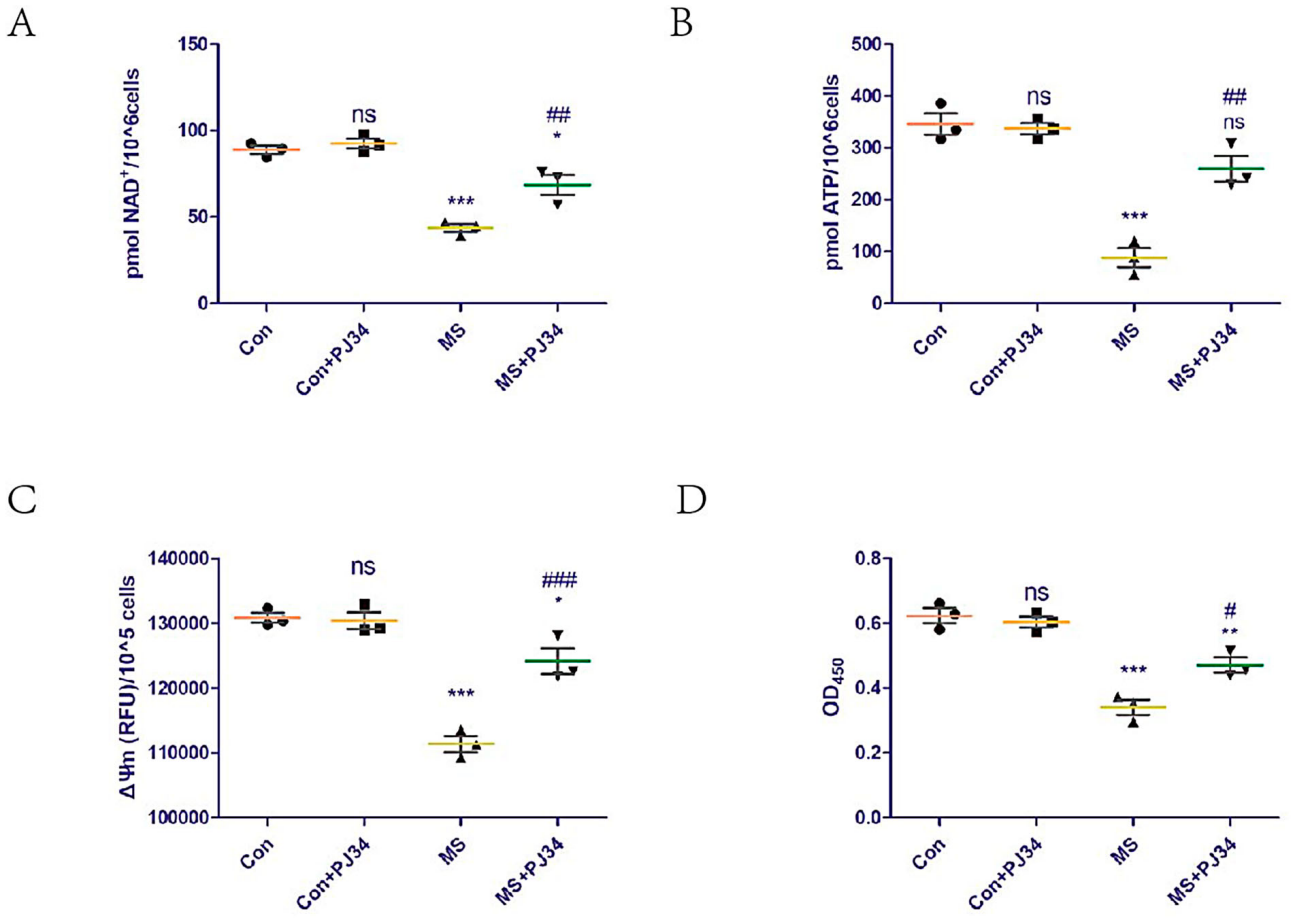
Inhibition of PARP-1 activity rescues cell damage induced by MS by increasing NAD^+^ levels and improving mitochondrial dysfunction. To inhibit PARP-1, C2C12 cells were treated with vehicle (PBS) or 1 μM PJ34 hydrochloride for 24 h before applying MS. (A) NAD^+^ levels in C2C12 cells detected using a commercial kit. (B and C) ATP content (B) and mitochondrial membrane potential (ΔΨm, C) were measured to assess mitochondrial function. (D) Cell viability was detected using the Cell Counting Kit-8 (CCK8) kit. All experiments were independently repeated at least three times. Statistical data are expressed as the mean ± standard deviation. Differences between the two groups were determined using Dunnett’s *t*-test. Tukey’s test was performed for multiple comparisons (compared to the control group: ***p < 0.001, **p < 0.01; *p < 0.05; ns, not significant; compared to the MS group: ^###^*p *< 0.001, ^##^*p* < 0.01; ^#^*p *< 0.05).

The effect of supplementation with exogenous PJ34 (a potent specific inhibitor of PARP-1) on NAD^+^ levels in C2C12 cells was assessed. The supplementation did not affect NAD^+^ levels in the control group but could rescue the NAD^+^ loss induced by mechanical overload (Figure 2A). In addition, we explored the effect of exogenous PJ34 supplementation on mitochondrial function in C2C12 cells. ATP content and ΔΨm are common indicators of mitochondrial function. Exogenous PJ34 supplementation did not affect the ATP content and ΔΨm of the control group but could rescue the mitochondrial dysfunction induced by mechanical overload (Figure 2B,C). The effect of exogenous PJ34 supplementation on C2C12 cell viability was also explored, as cell viability is a common indicator of cellular damage. Exogenous PJ34 supplementation did not affect the loss of cell viability of the control group but could rescue the cell damage induced by mechanical overload (Figure 2D).

### NMN supplementation rescues cell damage induced by mechanical overload by increasing NAD+ levels and improving mitochondrial dysfunction

**Figure 3.**
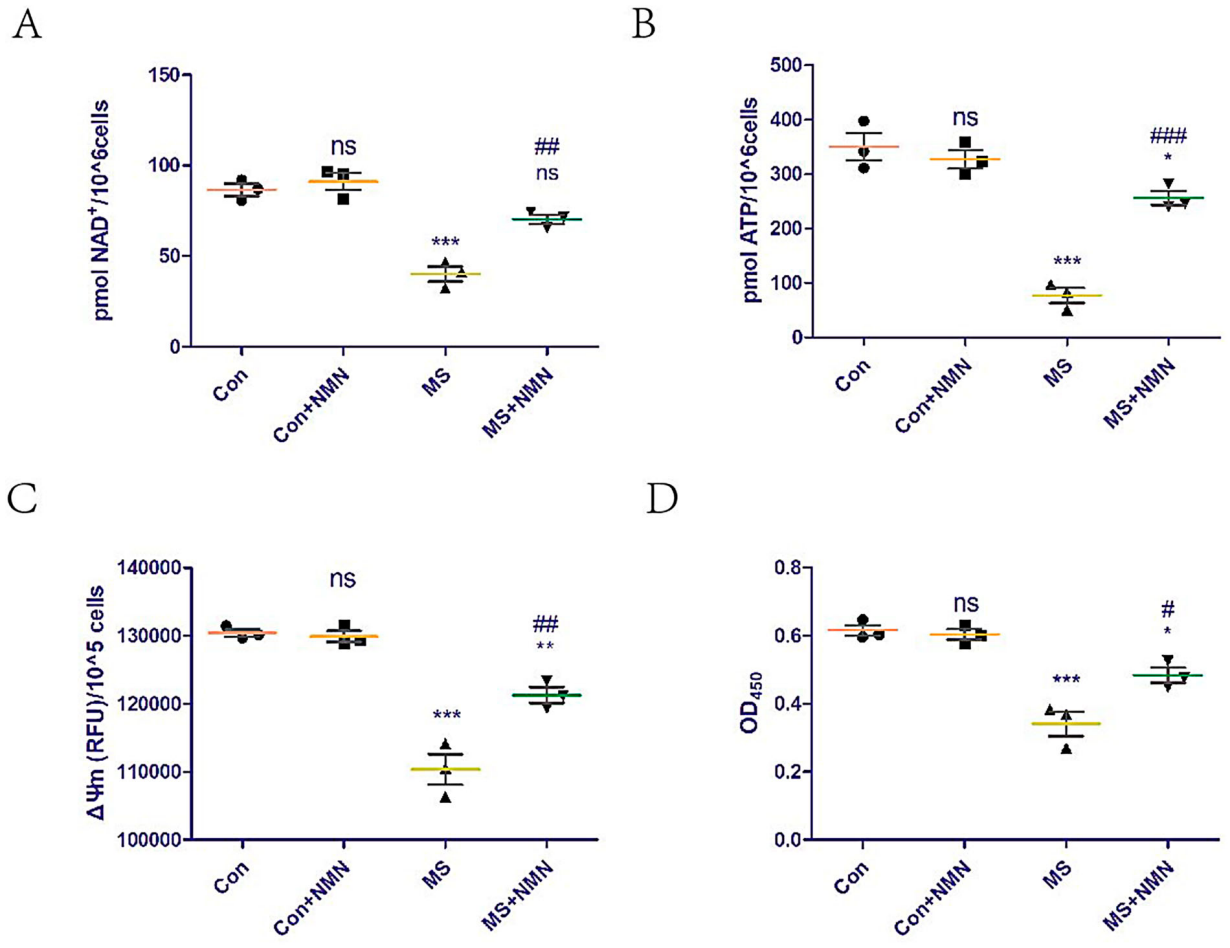
NMN supplementation rescues cell damage induced by mechanical overload by increasing NAD^+^ levels and improving mitochondrial dysfunction. To evaluate the effect of NMN supplementation, C2C12 cells were treated with vehicle (PBS) or 500 μM NMN for 24 h before applying MS. (A) NAD^+^ levels in C2C12 cells detected using a commercial kit. (B and C) ATP content (B) and mitochondrial membrane potential (ΔΨm, C) were measured to assess mitochondrial function. (D) Cell viability was detected using the Cell Counting Kit-8 (CCK8) kit. All experiments were independently repeated at least three times. Statistical data are expressed as the mean ± standard deviation. Differences between the two groups were determined using Dunnett’s *t*-test. Tukey’s test was performed for multiple comparisons (compared to the control group: ***p < 0.001, **p < 0.01; *p < 0.05; ns, not significant; compared to the MS group: ^###^*p *< 0.001, ^##^*p *< 0.01; ^#^*p *< 0.05).

The effects of supplementation with exogenous NMN (the most direct precursor of NAD^+^) on NAD^+^ levels in C2C12 cells was assessed. Exogenous NMN supplementation did not affect NAD^+^ levels in the control group but could rescue the NAD^+^ loss induced by mechanical overload (Figure 3A). We also explored the effect of exogenous NMN supplementation on mitochondrial function in C2C12 cells. Exogenous NMN supplementation did not affect the ATP content and ΔΨm of the control group but could rescue the mitochondrial dysfunction induced by mechanical overload (Figure 3B,C). Furthermore, exogenous NMN supplementation did not affect the C2C12 cell viability of the control group but could rescue the cell damage induced by mechanical overload (Figure 3D).

### Excessive mechanical traction causes breakage of DNA strands, increases PARP-1 activity, and reduces levels of NAD^+^ in LAM tissue

**Figure 4.**
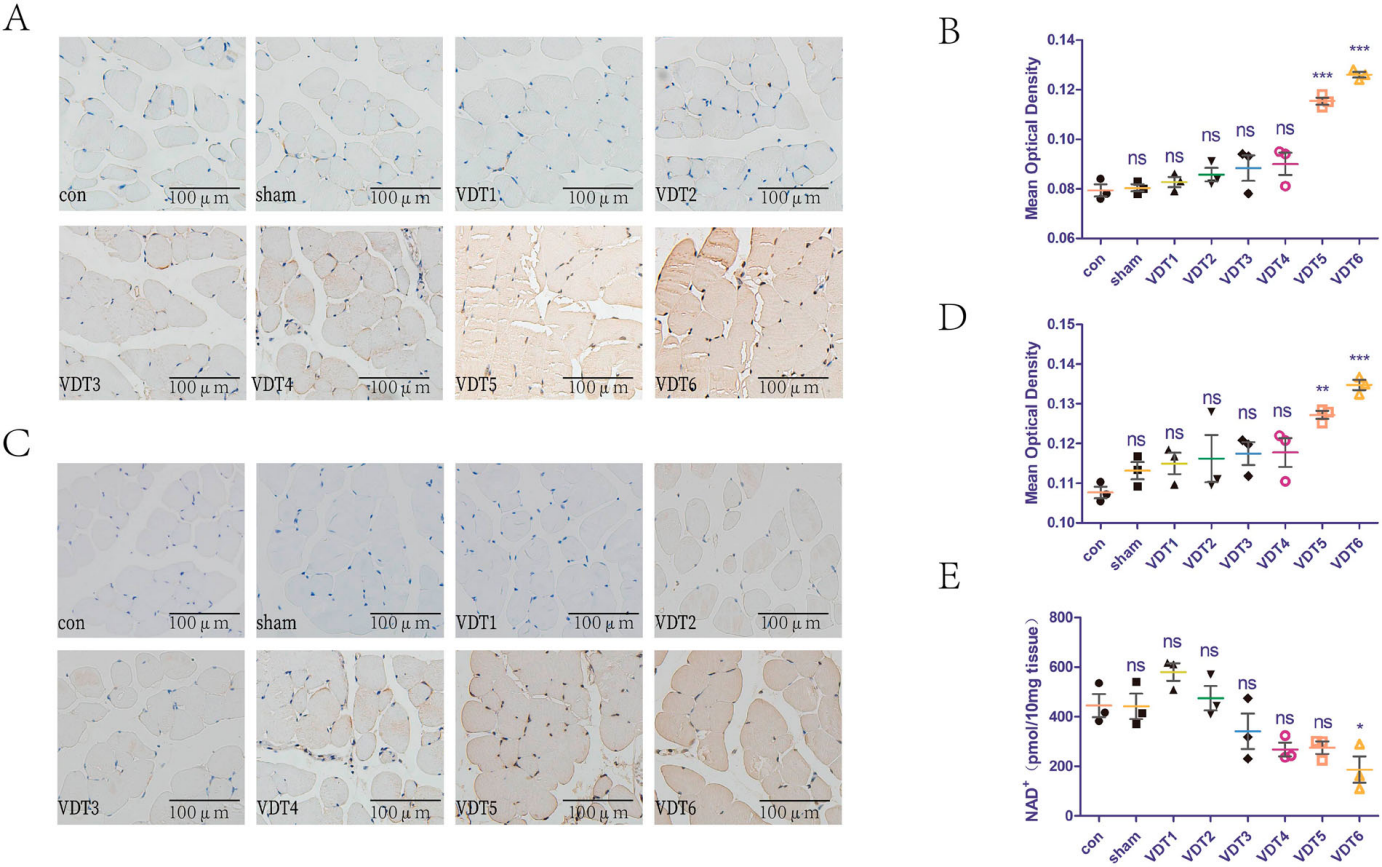
Excessive mechanical traction causes breakage of DNA strands, increases PARP-1 activity, and reduces levels of NAD^+^ in LAM tissue. (A and B) DNA damage of LAM was detected with immunohistochemistry staining of γH2AX. Mean optical density was semi-quantified using Image J software. (C and D) PARP-1 activity of LAM was detected with immunohistochemistry staining of PAR (C). Mean optical density (D) was semi-quantified using Image J. (E) NAD^+^ levels of LAM detected with a commercial kit. The experimental groups are detailed in the Material and Methods section. All experiments were independently repeated at least three times. The statistical data are expressed with the mean ± standard deviation. Differences between the two groups were determined using Dunnett’s *t*-test. Tukey’s test was performed for multiple comparison (compared with control group: ***p < 0.001, **p < 0.01; *p < 0.05; ns, not significant).

The effect of MS on the DNA of LAM tissues was investigated using different mechanical traction parameters. γH2AX is a common indicator of DNA damage. The mechanical traction parameters of VDT1-4 did not cause DNA damage, whereas those of VDT5-6 did (Figure 4A,B). In addition, we explored the effect of MS on PARP-1 activity and levels of NAD^+^ in LAM tissues. PAR is an indicator of PARP-1 activity. The mechanical traction parameters of VDT1-4 did not affect PARP-1 activity, while those of VDT5-6 resulted in PARP-1 overactivation (Figure 4C,D). Furthermore, the mechanical traction parameters of VDT1-5 did not affect the NAD^+^ levels of LAM tissue, while those of VDT6 reduced the NAD^+^ levels in the LAM tissue (Figure 4E).

### Inhibition of PARP-1 activity rescues LAM damage induced by MS by increasing NAD^+^ levels and improving mitochondrial dysfunction

**Figure 5.**
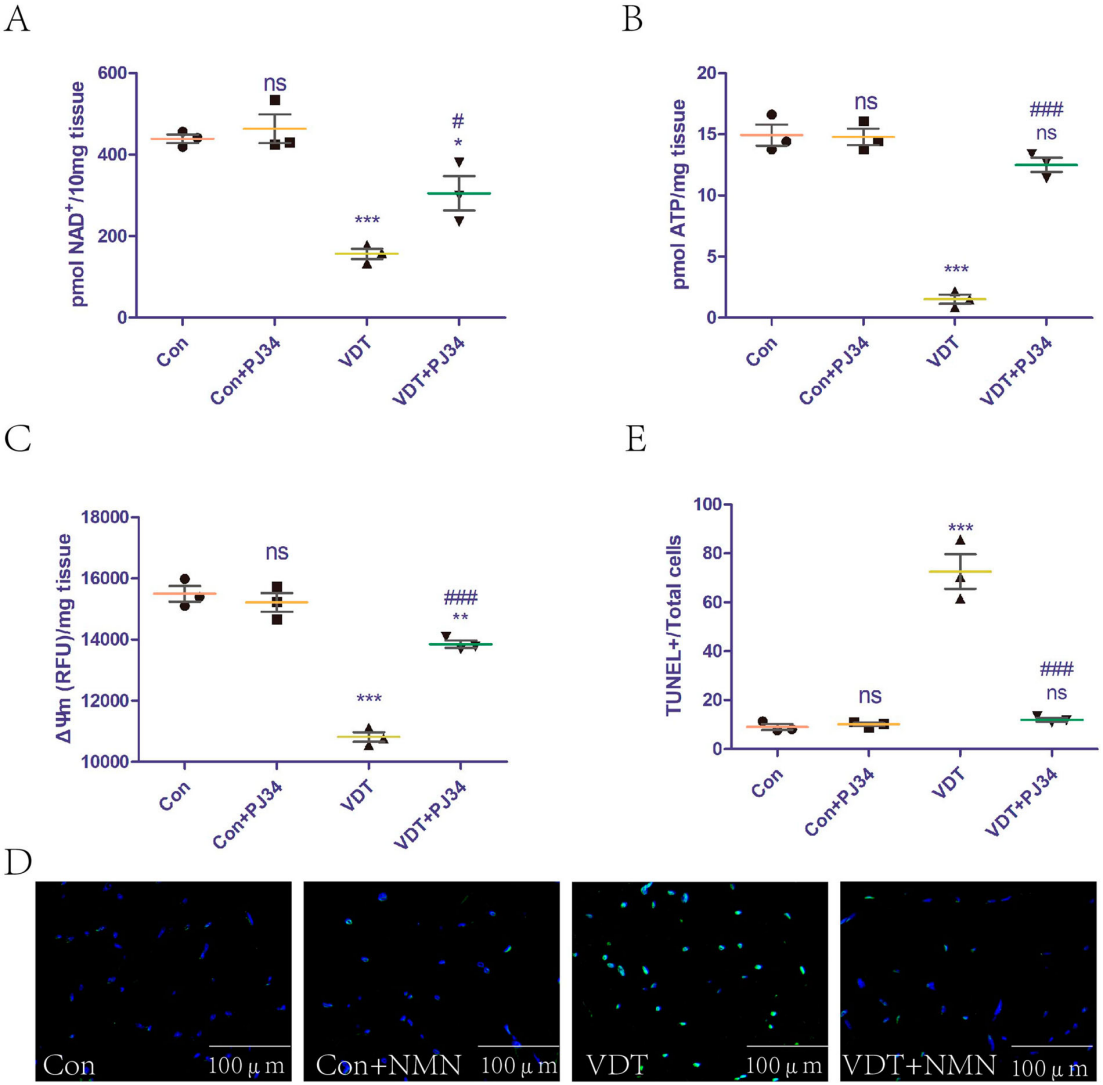
Inhibition of PARP-1 activity rescues LAM damage induced by MS by increasing NAD^+^ levels and improving mitochondrial dysfunction. To inhibit PARP-1, mice were intraperitoneally injected with vehicle (PBS) or 10 mg PJ34/kg body weight for 24 h before undergoing VDT. (A) NAD^+^ levels in the LAM detected using a commercial kit. (B and C) ATP content (B) and mitochondrial membrane potential (ΔΨm, C) were measured to assess mitochondrial function. (D and E) LAM damage was detected using tissue TUNEL staining. The positive staining area was semi-quantified using ImageJ software. All experiments were independently repeated at least three times. Statistical data are expressed as the mean ± standard deviation. Differences between the two groups were determined using Dunnett’s *t*-test. Tukey’s test was performed for multiple comparisons (compared to the control group: ***p < 0.001, **p < 0.01; *p < 0.05; ns, not significant; compared to the VDT group: ^###^*p *< 0.001, ^##^*p *< 0.01; ^#^*p *< 0.05).

The effect of exogenous PJ34 supplementation on NAD^+^ levels in LAM tissues was assessed. The supplementation did not affect NAD^+^ levels in the control group, but could rescue the NAD^+^ loss induced by excessive mechanical traction (Figure 5A). In addition, we explored the effect of exogenous PJ34 supplementation on mitochondrial function in LAM tissues. The supplementation did not affect the ATP content and ΔΨm of the control group, but could rescue the mitochondrial dysfunction induced by excessive mechanical traction (Figure 5B,C). The effects of exogenous PJ34 supplementation on LAM tissue damage were also explored. TUNEL staining is a common means to measure tissue damage. Exogenous PJ34 supplementation did not affect the LAM tissue of the control group, but could rescue the tissue damage induced by excessive mechanical traction (Figure 5D,E).

### NMN supplementation rescues LAM damage induced by MS through increasing NAD^+^ levels and improving mitochondrial dysfunction

**Figure 6.**
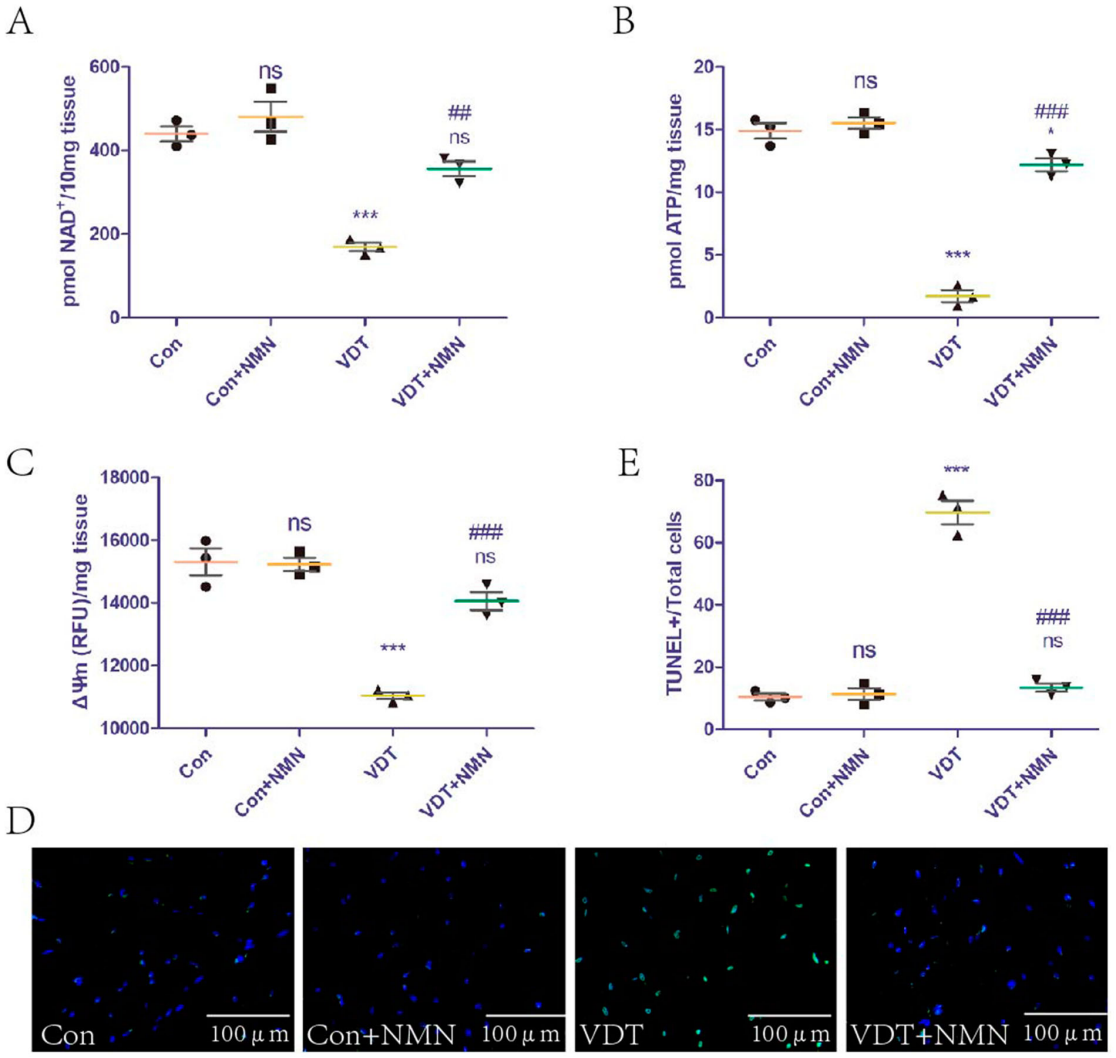
NMN supplementation rescues LAM damage induced by MS by increasing NAD^+^ levels and improving mitochondrial dysfunction.To evaluate the effect of NMN supplementation, the mice were intraperitoneally injected with vehicle (PBS) or 500 mg NMN/kg body weight for 7 consecutive days after undergoing VDT. (A) NAD^+^ levels in the LAM detected using a commercial kit. (B and C) ATP content (B) and mitochondrial membrane potential (ΔΨm, C) were measured to assess mitochondrial function. (D and E) LAM damage was detected using tissue TUNEL staining. The positive staining area was semi-quantified using ImageJ software. All experiments were independently repeated at least three times. Statistical data are expressed as the mean ± standard deviation. Differences between the two groups were determined using Dunnett’s *t*-test. Tukey’s test was performed for multiple comparisons (compared to the control group: ***p < 0.001, **p < 0.01; *p < 0.05; ns, not significant; compared to the VDT group: ###*p *< 0.001, ##*p* < 0.01).

The effect of exogenous NMN supplementation on NAD^+^ levels in LAM tissues was studied. The supplementation did not affect NAD^+^ levels in the control group, but could rescue the NAD^+^ loss induced by excessive mechanical traction (Figure 6A). In addition, we explored the effect of exogenous NMN supplementation on mitochondrial function in LAM tissues. The supplementation did not affect the ATP content and ΔΨm of the control group, but could rescue the mitochondrial dysfunction induced by excessive mechanical traction (Figure 6B,C). The effects of exogenous NMN supplementation on LAM tissue damage were also explored. The supplementation did not affect the LAM tissue of the control group, but could rescue the tissue damage induced by excessive mechanical traction (Figure 6D,E).

## Discussion

PFD is an international and socialized health problem that seriously affects the social, physical, and mental health of female patients. As the main tissue of the pelvic floor support structure, LAM is important in maintaining normal function of the pelvic floor organs. Irreversible LAM damage leads to PFD. In clinical practice, vaginal delivery is the main cause of PFD (Hendrix S et al. 2002; Mant et al. 1997; Rortveit et al. 2001; Swift et al. 2005). Compared to non-partum women, women who have given birth are more likely to develop PFD. Further, women who undergo vaginal delivery are more likely to develop PFD than those who undergo cesarean section (Blomquist et al. 2018). Mechanical trauma related to childbirth is important in this process (Shek et al. 2012). Vaginal delivery causes LAM to undergo varying degrees of stretching (Lien et al. 2004). According to muscle physiology, when skeletal muscle is stretched to more than 1.5 times its original length, its ultrastructure is severely damaged (Brooks et al. 1995). Therefore, it is not difficult to understand that childbirth-related mechanical trauma can lead to a variety of disorders.

We previously demonstrated that MS caused LAM damage in mice (Yi et al. 2020). The present study extended this observation by exploring the relevant mechanisms of MS-induced damage, with the aim of identifying therapeutic targets. NAD^+^ has important roles in muscle development, homeostasis, and various diseases by regulating mitochondrial function. After specifically deleting nicotinamide phosphoribosyl transferase, an essential enzyme in the NAD^+^ salvage synthesis pathway, knockout mice exhibit a dramatic decline in intramuscular NAD^+^ levels, accompanied by loss of muscle strength and fiber degeneration (Frederick et al. 2016). However, the role of NAD^+^ in the damage of both muscle cells and LAM tissue induced by MS has remained unclear. The present findings indicated that mechanical loading up to the parameters of MS4-6 caused DNA damage, and with those of MS5-6 caused overactivation of PARP-1. However, only mechanical loading up to the MS6 parameters reduced intracellular NAD^+^ levels (Figure 1). These findings indicate that a small amount of DNA damage and PARP-1 activation does not affect intracellular NAD^+^ levels, whereas excessive DNA damage and PARP-1 activation leads to an imbalance in cellular NAD^+^ homeostasis. Animal experiments reached the same conclusion (Figure 4).

This study further explored the mechanism of NAD^+^ in the damage of both muscle cells and LAM tissue induced by MS. The maintenance of NAD^+^ homeostasis depends on the balance between NAD^+^ degradation and biosynthesis. In this study, exogenous addition of PARP-1 inhibitors (which inhibit NAD^+^ degradation) and NMN (which promote NAD^+^ biosynthesis) was used to increase NAD^+^ levels. Increasing NAD^+^ levels *in vivo* and *in vitro* could rescue mitochondrial dysfunction and damage to both muscle cells and LAM tissue induced by MS. However, exogenous addition of PARP-1 inhibitor and NMN had no effect on normal muscle cells and LAM tissue. This might be because normal cells and tissues have the ability to regulate NAD^+^ homeostasis, whereas mechanical damage causes cells and tissues to lose this ability. Similarly, increasing NAD^+^ levels can alleviate or even reverse age-related mitochondrial damage in skeletal muscles (Mendelsohn and Larrick 2019; Dao et al. 2020).

There are several limitations of this study. We only studied the effects of NAD^+^ on the damage to both muscle cells and LAM tissue induced by MS at the cellular and animal levels. As human LAM tissue is difficult to obtain, we lack data on human-derived LAM tissue. In addition, the study lacked relevant information on how NAD^+^ regulates mitochondrial function. In aging muscles of rats, mice, and humans (Gomes et al. 2013; Massudi et al. 2012; Braidy et al. 2011), low levels of NAD^+^ will lead to decreased Silent information regulator 1 (SIRT1) activity and imbalance of mitochondrial homeostasis. Feeding NAD^+^ precursors can significantly increase SIRT1 activity in young and old mice, and improves oxidative metabolism (Mills et al. 2016; Pajk et al. 2017). These findings indicate that NAD^+^ may regulate mitochondrial function by affecting the activity of SIRT1. We intend to explore this issue in future studies.

In conclusion, MS induces damage in both C2C12 cells and LAM tissue. Restoring NAD^+^ homeostasis rescues the damage by improving mitochondrial function.

## Data Availability

The analyzed datasets generated during the study are available from the corresponding author upon reasonable request.
